# Supplementary material for: Practicality of training a quantum-classical machine in the noisy intermediate-scale quantum era
Source: iScience. 2025 Jul 9;28(8):113058. doi: 10.1016/j.isci.2025.113058 (PMC12312051; doi:10.1016/j.isci.2025.113058)
Supplement: Document S1. Figures S1–S17 [file mmc1.pdf]

iScience, Volume *28*

Supplemental information

Practicality of training
a quantum-classical machine
in the noisy intermediate-scale quantum era

Tarun Dutta, Alex Jin, Clarence Liu Huihong, José Ignacio Latorre, and Manas Mukherjee

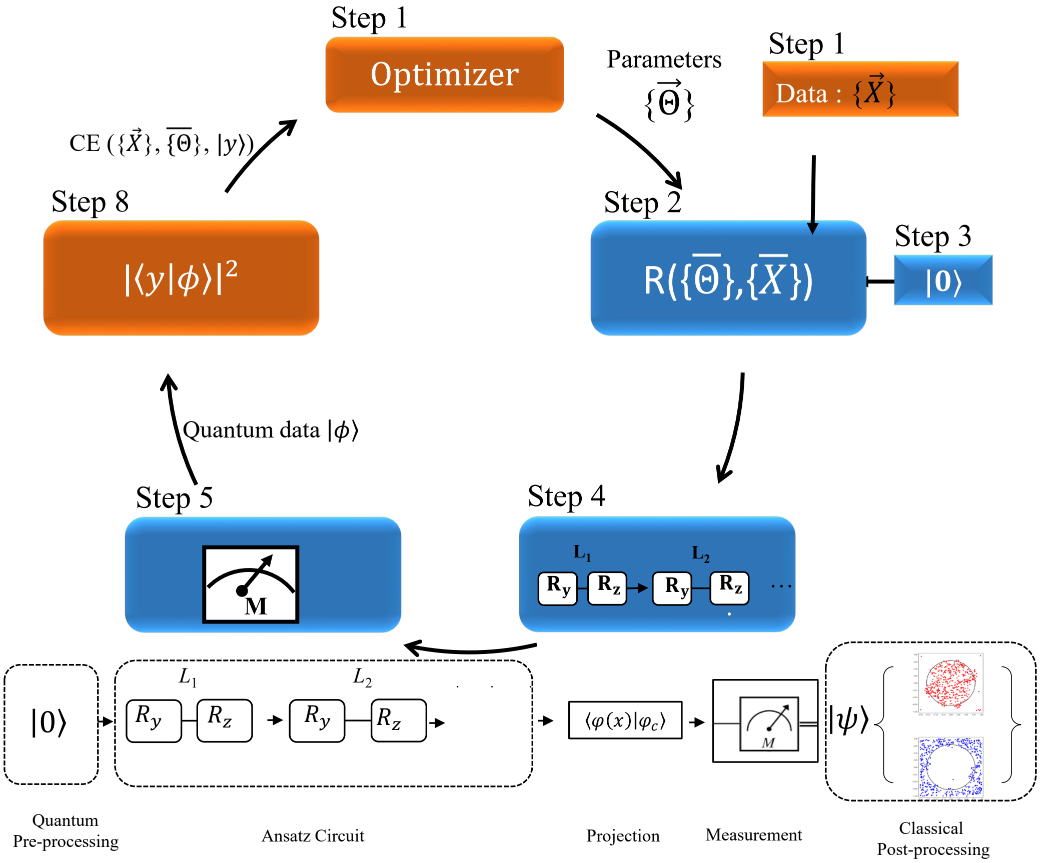

FIG. S1. Training: this concise circular representation highlights the quantum training process for classification using classical and quantum components. The circular flow illustrates the path of data and operations, starting with classical pre-processing, moving to quantum processing, and concluding with classical post-processing and model updates. Arrows indicate the direction of data flow and processing steps. The key points in this schematic are following: *Classical pre-processing:* The CPU pre-processes the training data, and the resulting features are used as input to the quantum circuit.*Classical to quantum interface (Encode)*: The classical data is encoded into a quantum gate using DDS and FPGA components (*Quantum hardware*) to implement precise quantum control signals and quantum gates.*Quantum circuit*: The QPU executes a quantum circuit to perform the classification task using the quantum-encoded data. *Quantum to classical interface (Decode)*: The quantum results are decoded back into classical data using PMT and FPGA. *Classical post-processing*: The CPU processes the classical results, performs analysis, and updates the model parameters based on the quantum data.

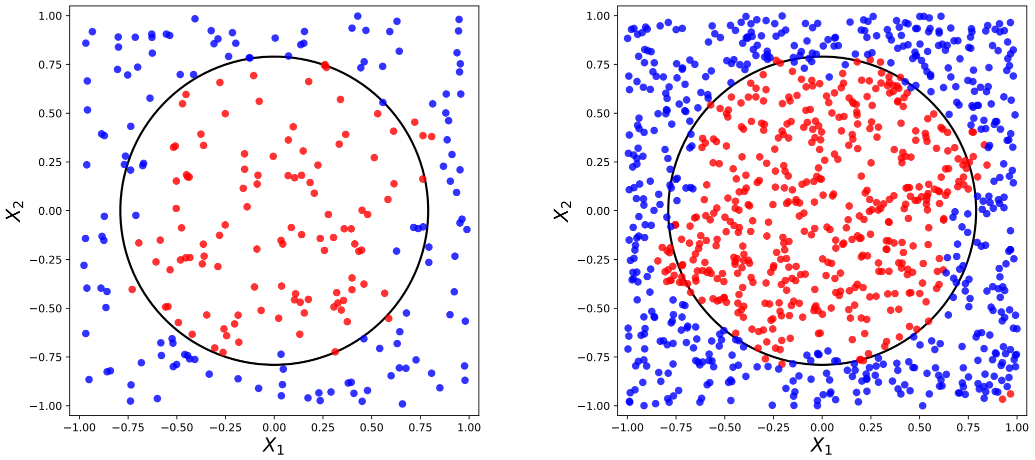

FIG. S2. Training and validation: based on the best parameters obtained after 12 generations (training 2 of fig. 2c in the main text) using the Genetic Algorithm (GA) optimizer, the classification results for a training dataset (left) comprising 250 points and a test dataset (right) consisting of 1000 points are depicted in this figure. The achieved accuracy of the classification on the test dataset is approximately $92.8 \pm 1.8\%$, while the training accuracy reached $93.6 \pm 1.8\%$ for a before-after comparison of the training procedure).

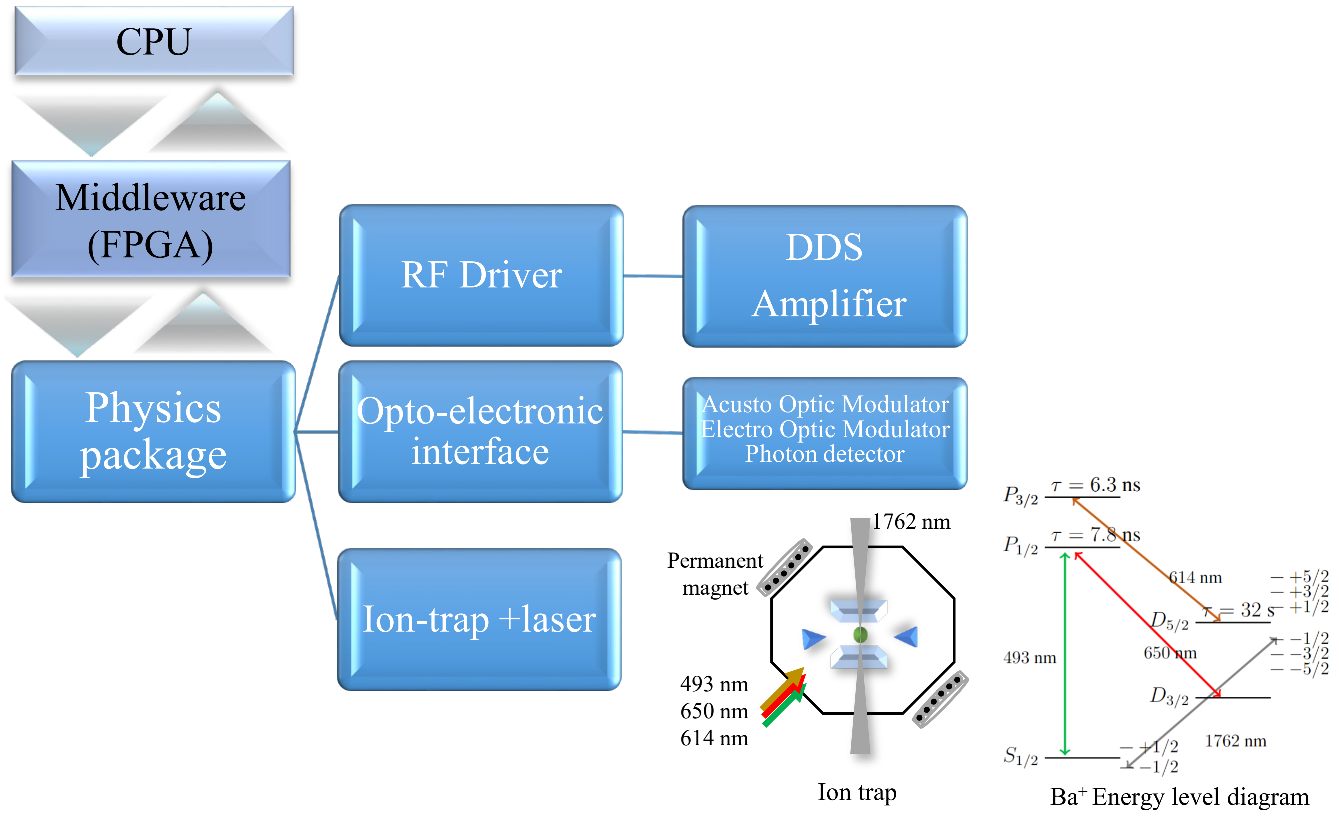

FIG. S3. The full-stack hybrid quantum-classical system comprises of three main layers: physics package or the quantum processing unit (QPU), middleware, and CPU. The QPU layer encompasses the ion trap setup along with lasers, the opto-electronic interface and the RF-drivers. The middleware is the interface between the QPU and the CPU and realized by a FPGA. The top-most layer is the CPU which implements the optimizer in a Linux environment using python script. In our ion trap setup, we specifically work with barium ions. The energy level diagram of barium ions, shown in the schematic, provides crucial information about the lasers involved in cooling, measurement, and gate implementation. Further details are discussed in the text.

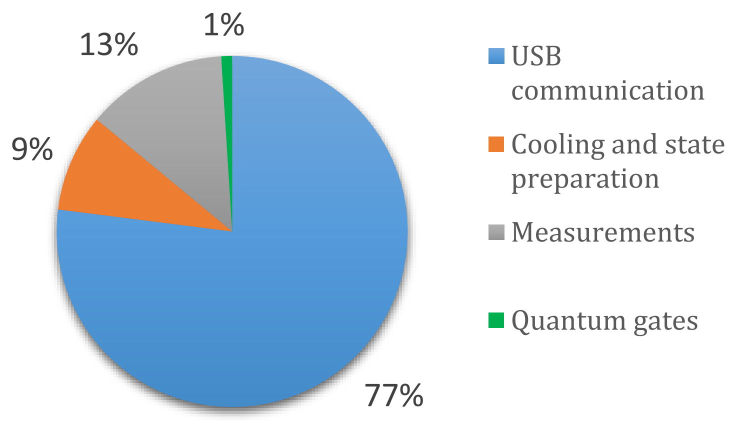

FIG. S4. Time Budget: the time required to complete the training of one generation for a training dataset of 250 data points, with a set of 50 individuals, is approximately 330 min in our current setup. Each data point in the training dataset is iterated 150 times. The distribution of time for each step is depicted in the PI-chart. A significant portion of the time is allocated to USB serial communication, which mainly involves loading data onto the Field-Programmable Gate Array (FPGA), loading data onto the Direct Digital Synthesis (DDS), and receiving data from the FPGA. The remaining time is dedicated to tasks related to cooling, measurement, and gate implementation.

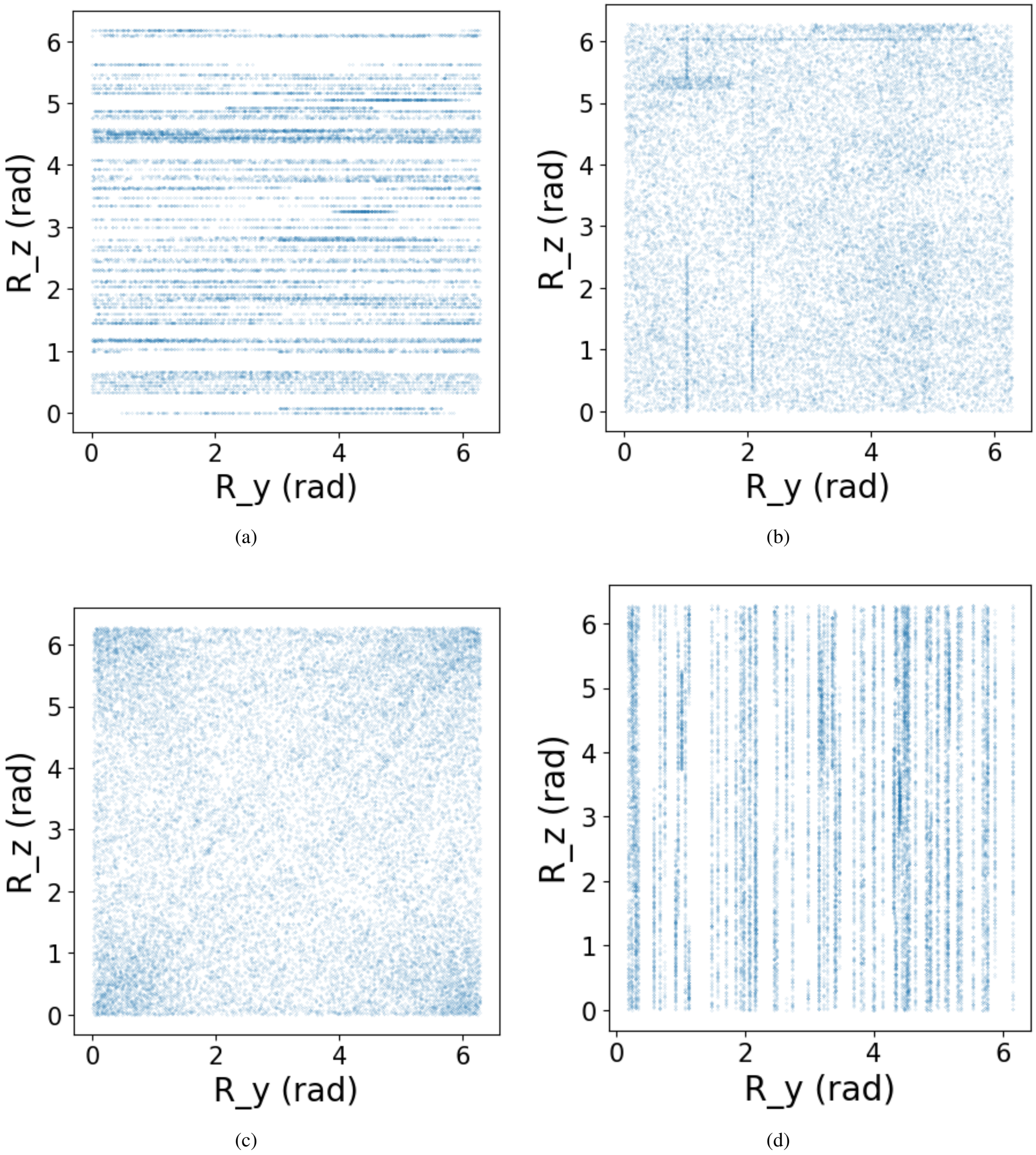

FIG. S5. Choice op kernel: Aggregate rotational values of 20 randomly chosen sets of parameters using different Ansatz. (a) Ansatz 2A. (b) Ansatz 2B. (c) Ansatz 2C. (d) Ansatz 2D. See the section *choice of kernal* in the main text method section.

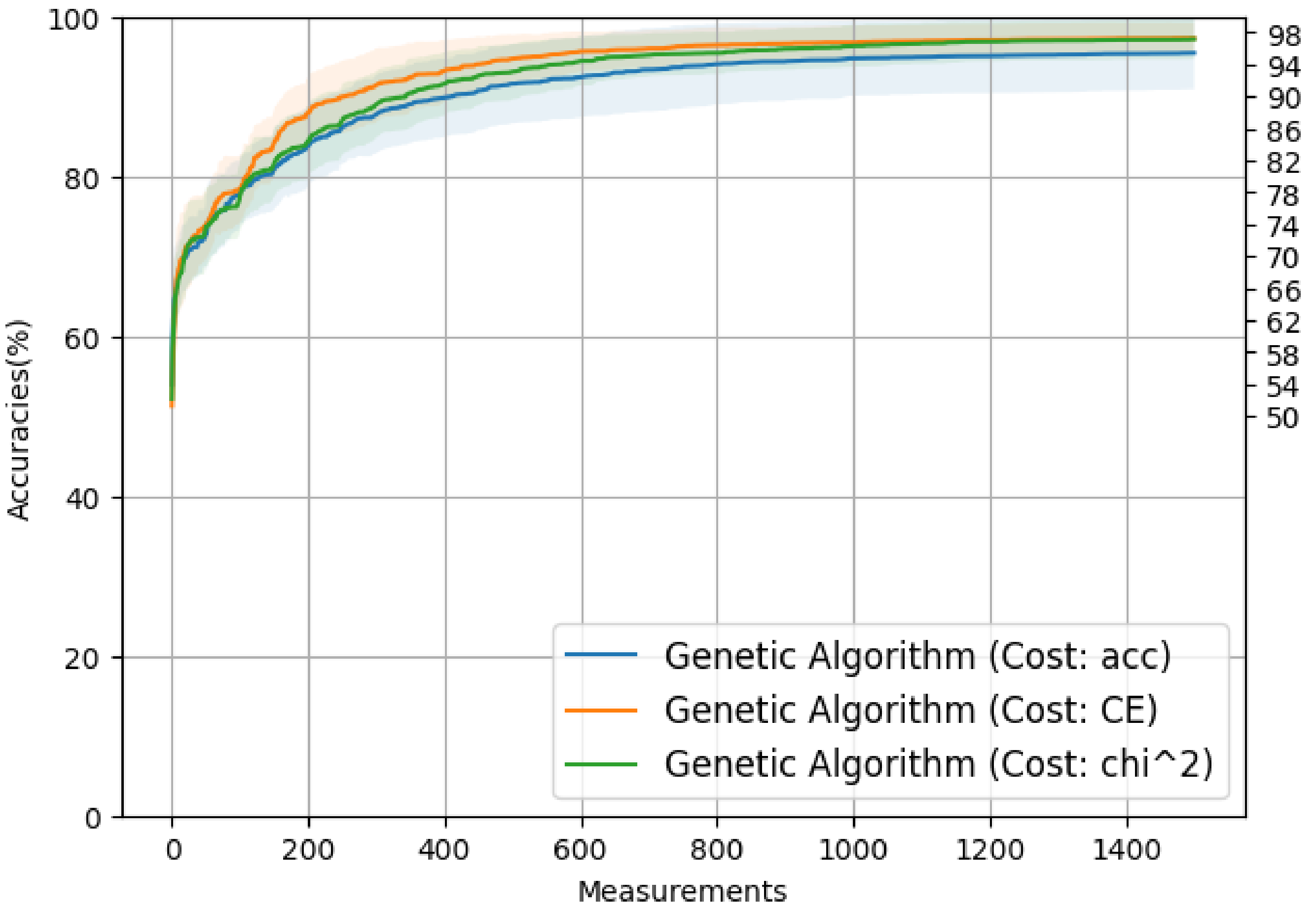

FIG. S6. Simulation results of highest cumulative GA accuracy with three different metrics. Cross-entropy loss gives best rate of convergence. Both cross-entropy and chi squared loss shows comparable final accuracy. Using accuracy as loss shows both low converged value and slower rate of convergence.

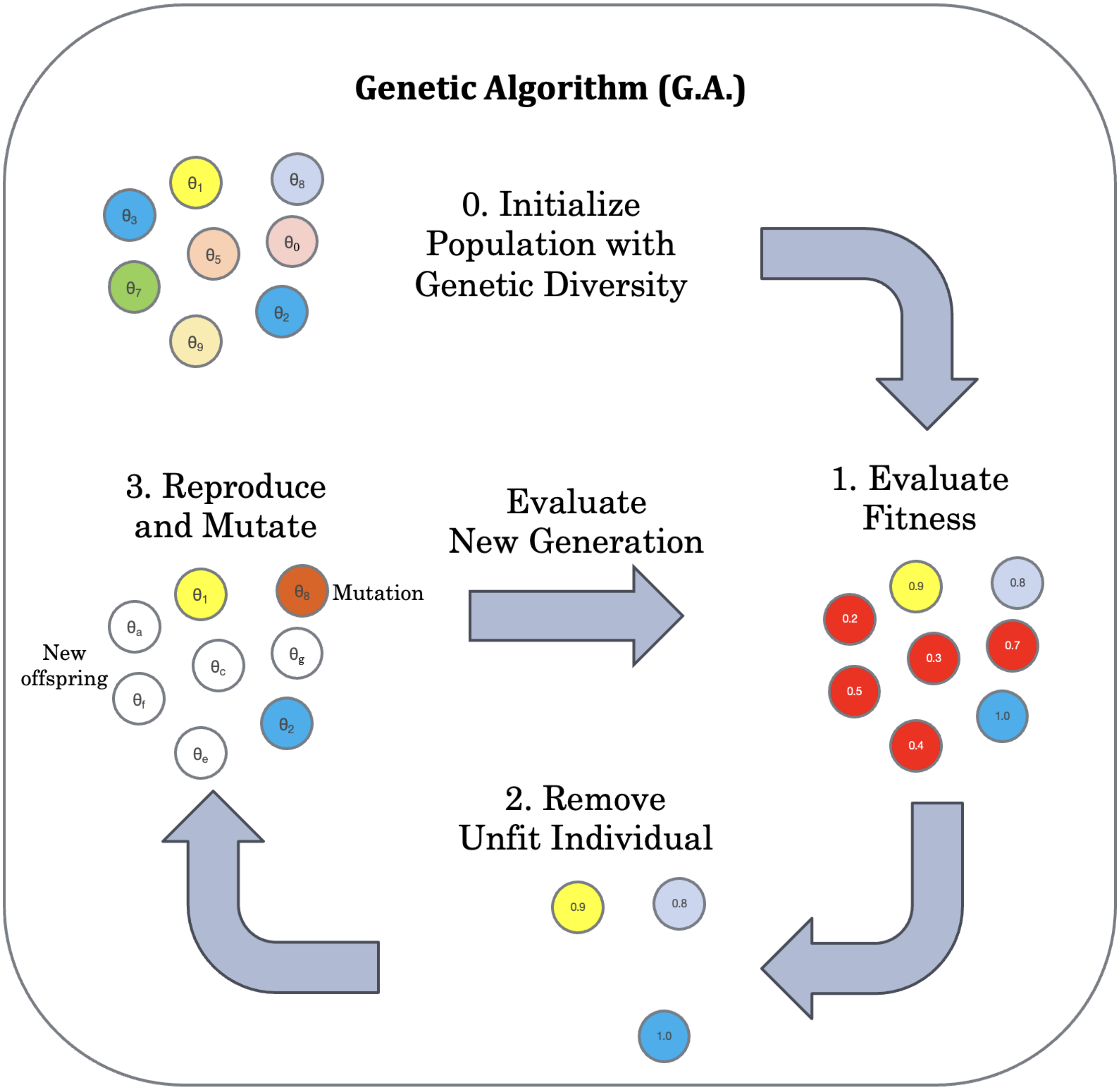

FIG. S7. Life-cycle of genetic algorithm. This serves as a visual illustrate to Algorithm **??**.

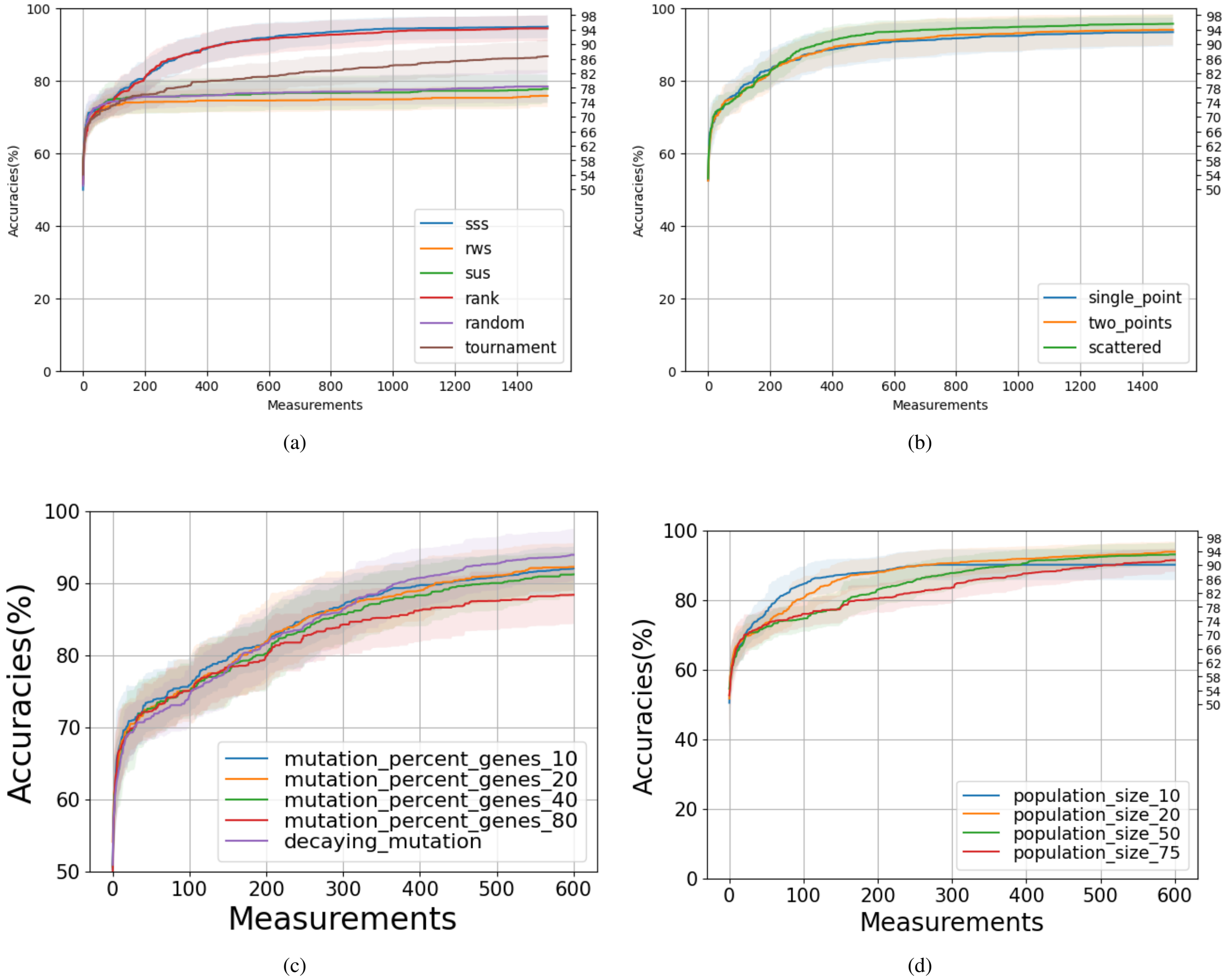

FIG. S8. Simulation results on various GA hyper-parameters. For all plots, cumulative highest accuracy is plotted against measurements. (a): comparison of GA with steady state selection (sss), roulette wheel selection (rws), stochastic universal selection (sus), rank selection (rank), random selection (random), and tournament selection (tournament). (b): comparison of GA with single point, two points, and scattered crossover. (c): comparison of GA with 10%, 20%, 40%, 80% fixed mutation rates, and decaying mutation. (d): comparison of GA with 10, 20, 50, and 75 population pool.

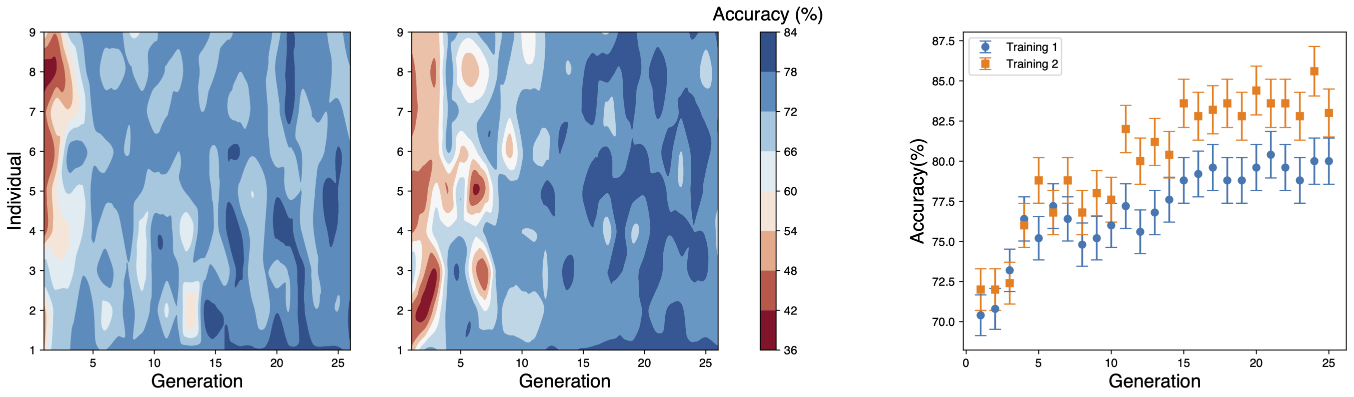

FIG. S9. *Left*: Training run 1. *Center*: Training run 2. *Right*: Search for optimal parameters for classification task using Genetic algorithm. The ion trap-based QPU is used for training on 250 random data points. The depth of the circuit is kept fixed to 4 layers as it is sufficient to classify the current problem. For this training, a set of 9 individuals have been used for training and the data has been uploaded using ansatz 2A.

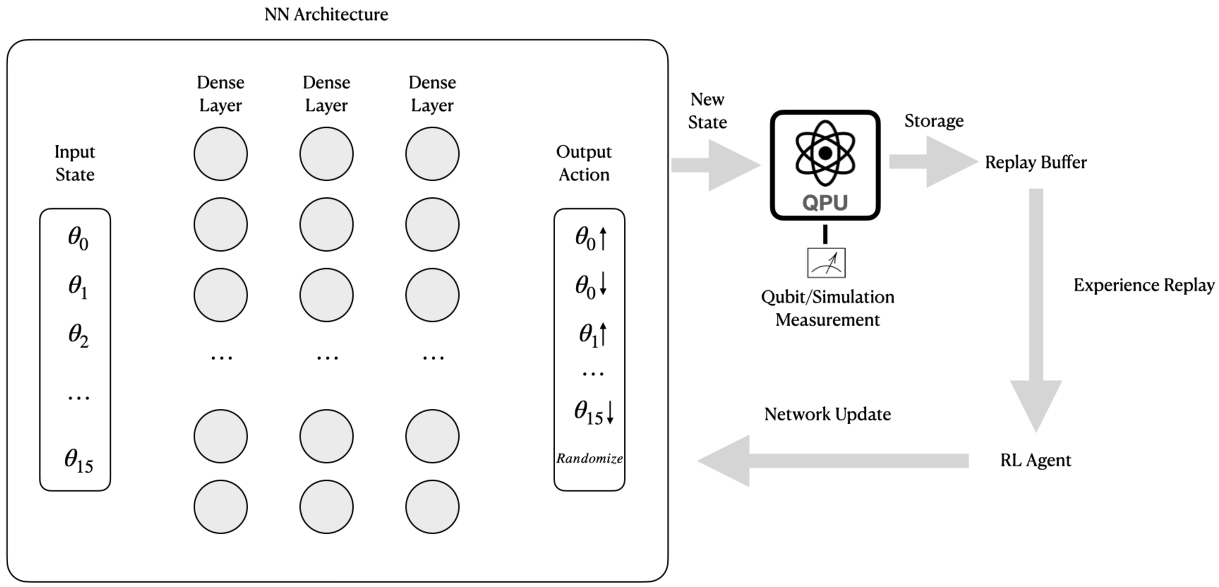

FIG. S10. Deep Reinforcement Learning (RL) Architecture used as a training agent for the quantum classifier. The values of our trainable parameters are the states of our RL problem. There are 4n+1 actions available for the agent to select from. For each parameter, we have 4 actions: increment, decrement, tiny increment, and tiny decrement. Finally we have a *randomize* action that either picks a completely random value, or reset to the initial value depending on the training paradigm. The new state is measured on the simulation, and the reward (increase in accuracy/cross-entropy is stored in a replay buffer. The agent then randomly samples mini-batches of tuples (state, action, next state, reward) for training. The network is updated after each training step.

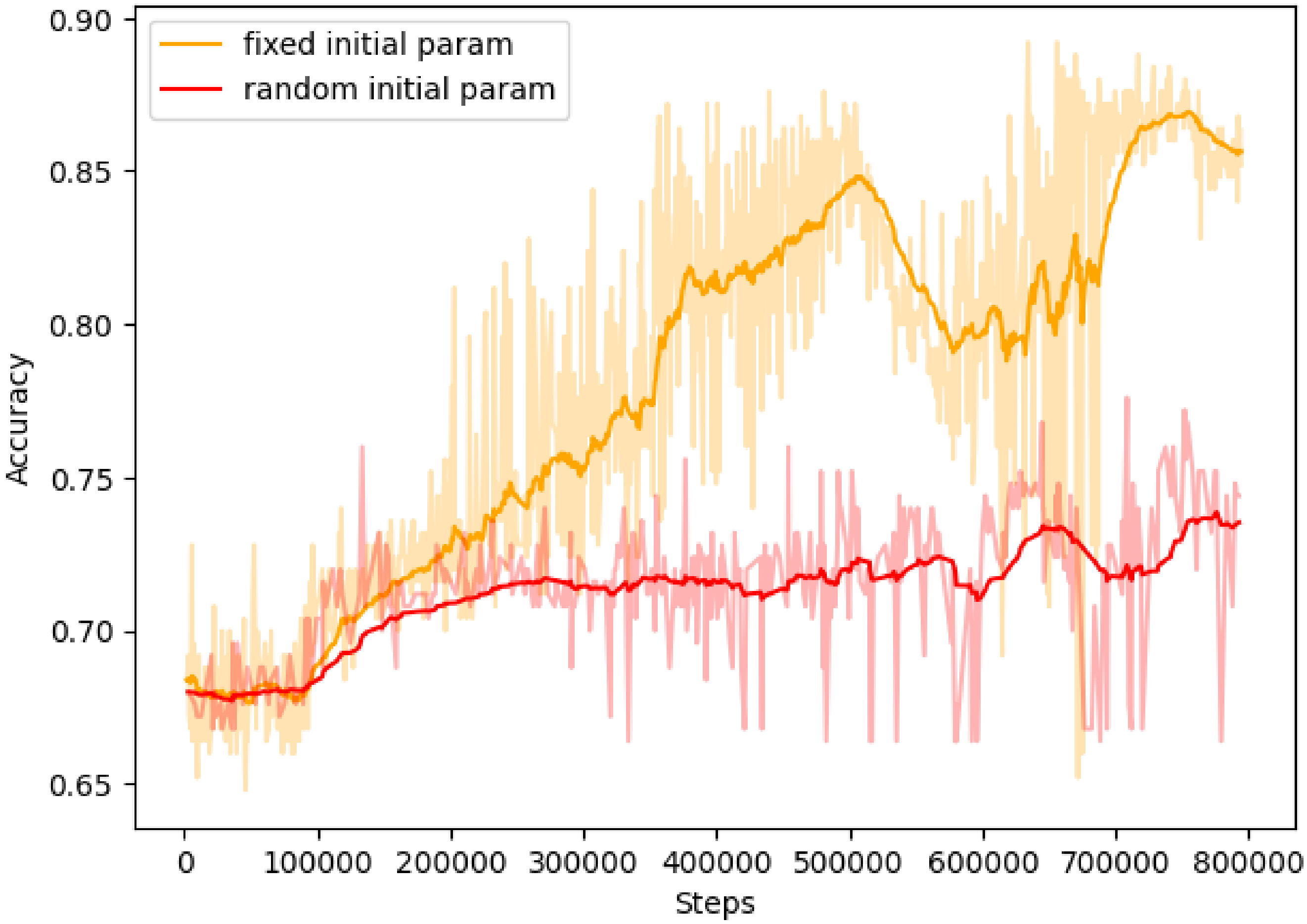

FIG. S11. Each step represents an agent taking 500 actions. Highest training accuracy achieved within 500 iterations is recorded as a single data point. The plot shows two training runs each with around 800,000 training steps. Faint lines represent the raw data and solid lines represent exponentially smoothed values with a smoothing factor of 0.95.

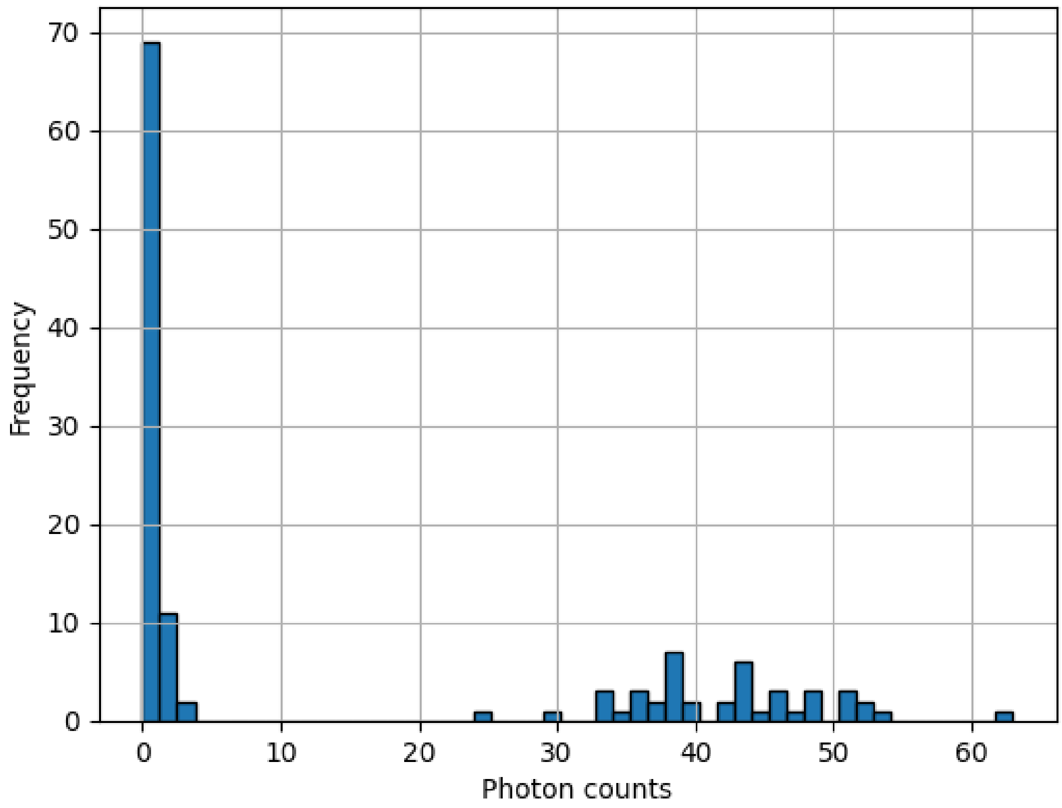

FIG. S12. Histogram of 125 qubit projection shots. $|0\rangle$: photon counts between 0 and 12, and $|1\rangle$ photon counts greater than 12. Measured $\alpha^2 = 0.656$ and $\beta^2 = 0.344$.

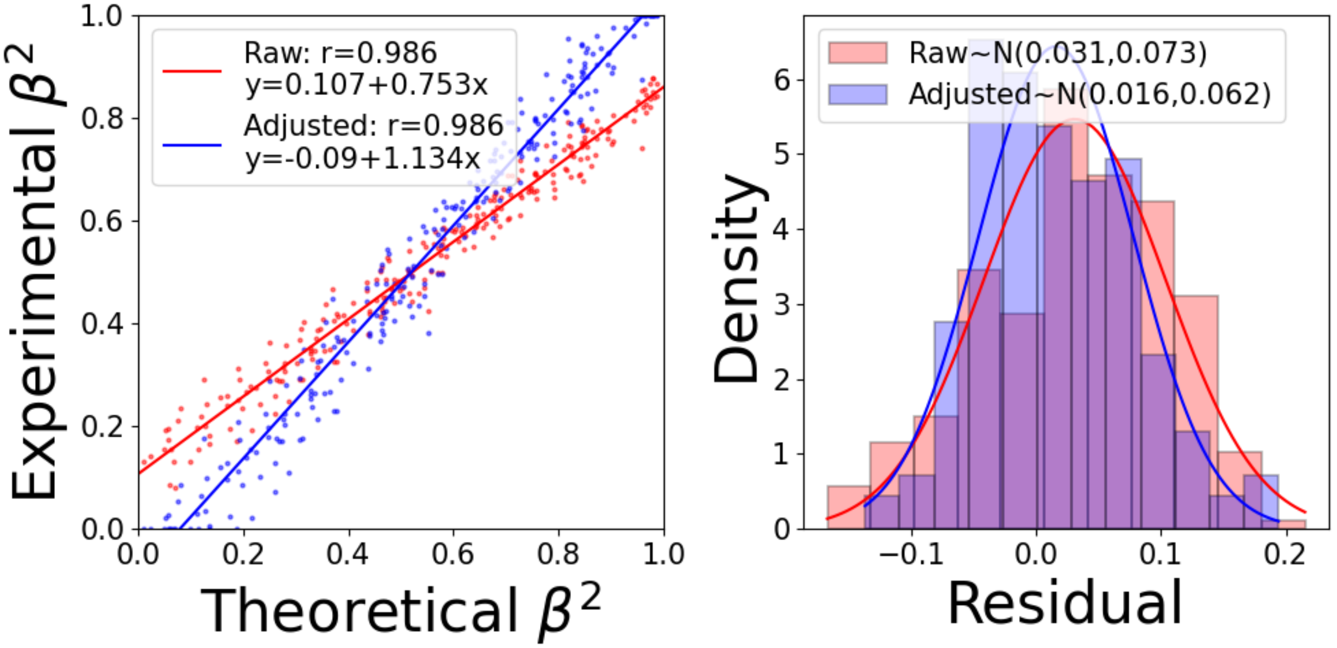

FIG. S13. Left: repeated results from Figure (S12) over the entire training dataset and plot experimental against theoretical β^2 values. Post-application of inverse correlation matrix to experimental values is displayed in blue. Right: residuals (theoretical value - experimental value) of the results from the left. The histogram is fitted with a normal distribution and the y-axis is normalized using Gaussian density function.

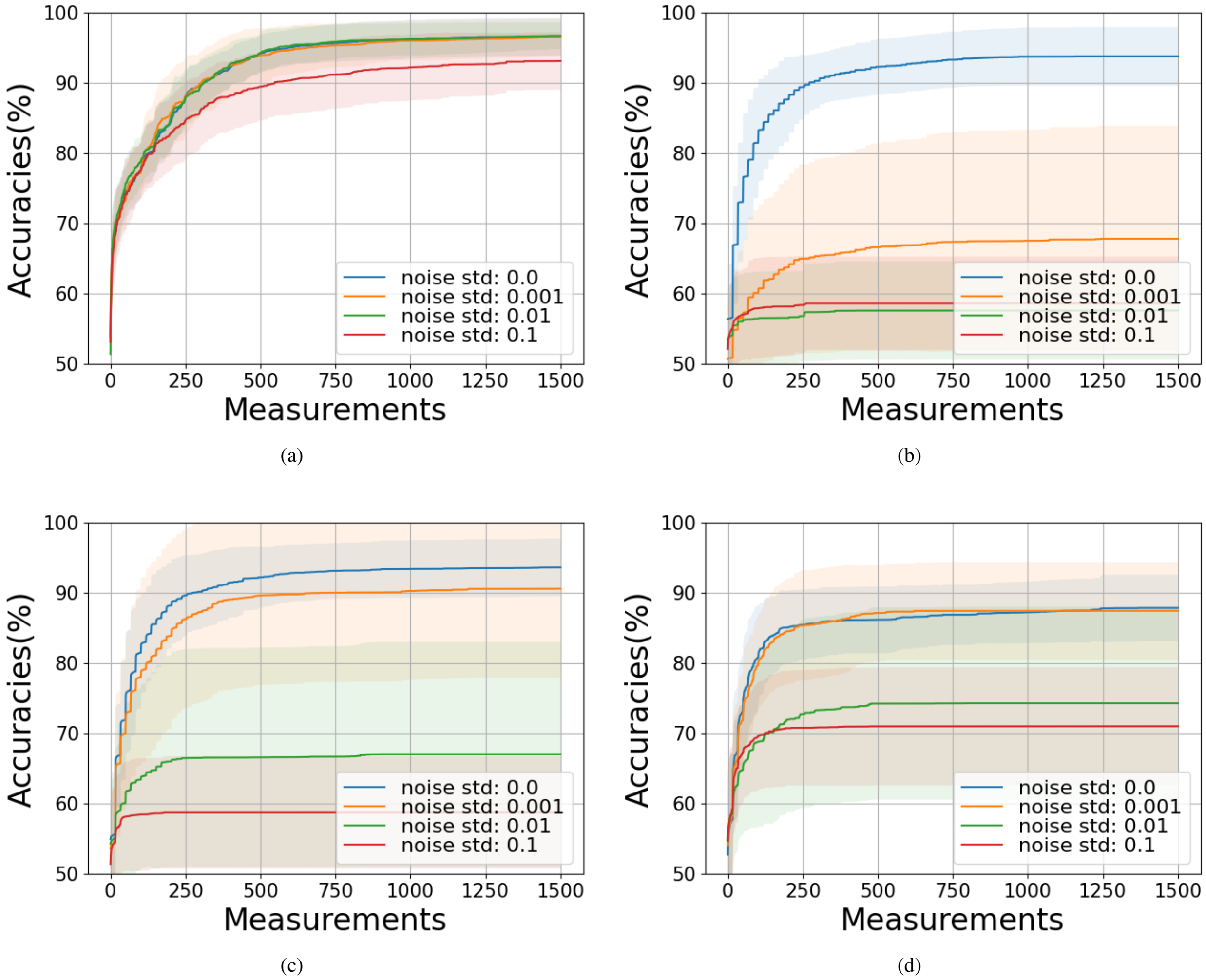

FIG. S14. Simulation of binary classification problem solved with a 4-layer data re-uploading architecture and varying Gaussian noise levels. All noises are assumed to have 0 mean. (a) Genetic Algorithm solver with population size 50, scattered crossover, exponentially decaying mutation rate, and sss parent selection scheme. (b) l-BFGS-b solver with stepsize (for gradient estimation) of 0.005. (c) l-BFGS-b solver with stepsize (for gradient estimation) of 0.05. (d) l-BFGS-b solver with stepsize (for gradient estimation) of 0.5.

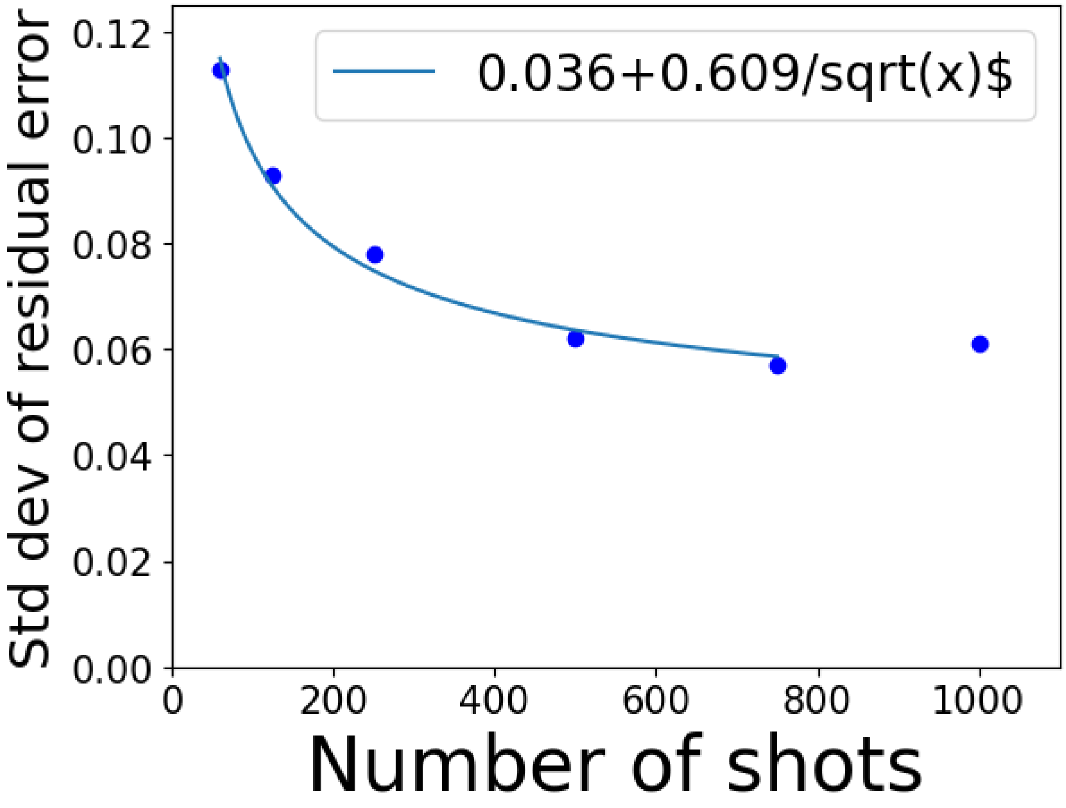

FIG. S15. Standard deviation of residual errors (see example Figure S13) computed with different number of repetitions between 60 and 1000. The curve is fitted against the first 5 points.

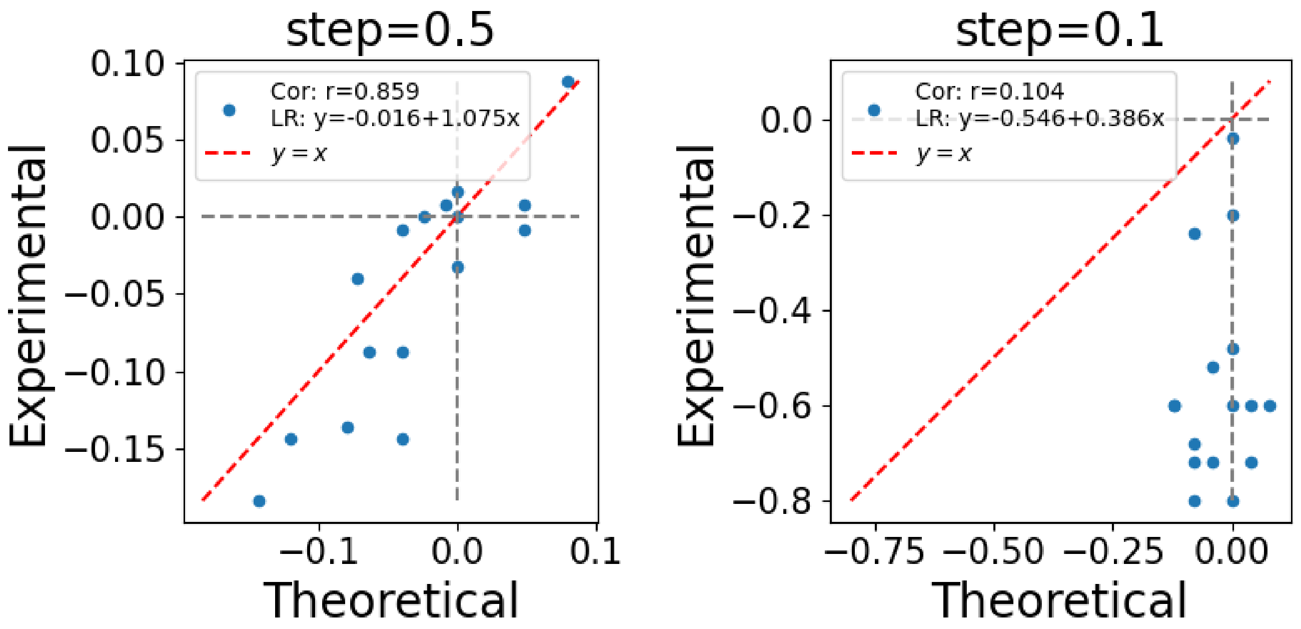

(a) (b)

FIG. S16. Experimental vs theoretical gradients using three different metrics. (a) Gradient computed using step size of 0.5. Dashed lines represent the x and y axes. Points in quadrants 2 and 4 represent experimental and theoretical gradients having opposite signs. (b) Gradient computed using step size of 0.1.

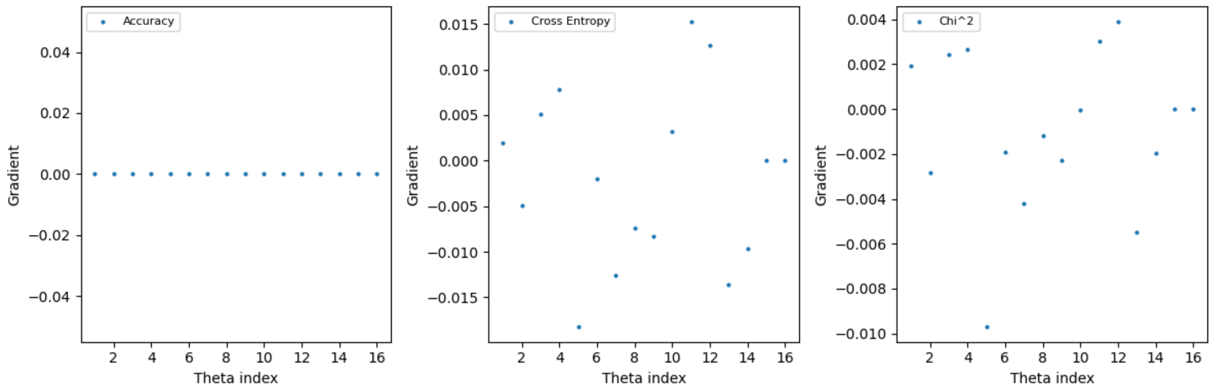

FIG. S17. Partial derivative of Accuracy, Cross-entropy, Chi-squared values (see *cost-functions* in the main text *method* section) using finite difference with step size of 0.01.
